# Towards Personalized Treatment in Haemophilia: The Role of Genetic Factors in Iron and Heme Control to Identify Patients at Risk for Haemophilic Arthropathy

**DOI:** 10.3390/jpm14020145

**Published:** 2024-01-28

**Authors:** Lize F. D. van Vulpen, Simon C. Mastbergen, Wouter Foppen, Kathelijn Fischer, Floris P. J. G. Lafeber, Roger E. G. Schutgens

**Affiliations:** 1 Centre for Benign Hematology, Thrombosis and Haemostasis, Van Creveldkliniek, University Medical Center Utrecht, University Utrecht, Postbus 85500, 3508 GA Utrecht, The Netherlands; k.fischer@umcutrecht.nl (K.F.); r.schutgens@umcutrecht.nl (R.E.G.S.); 2 Department of Rheumatology & Clinical Immunology, University Medical Center Utrecht, University Utrecht, 3508 GA Utrecht, The Netherlands; s.mastbergen@umcutrecht.nl (S.C.M.); f.lafeber@umcutrecht.nl (F.P.J.G.L.); 3 Department of Radiology and Nuclear Medicine, Division of Imaging & Oncology, University Medical Center Utrecht, University Utrecht, 3508 GA Utrecht, The Netherlands

**Keywords:** haemophilia, arthropathy, personalized medicine, *HFE*, *HMOX1*

## Abstract

The treatment landscape for haemophilia is changing rapidly, creating opportunities for personalized treatment. As major morbidity is still caused by haemophilic arthropathy, understanding the factors affecting joint damage and joint damage progression might lead to more individualized treatment regimens. We investigated the association of *HFE* mutations or *HMOX1* polymorphisms affecting iron/heme handling with radiographic joint damage in 252 haemophilia patients (severe and moderate). Although iron levels and transferrin saturation were significantly increased in the 95 patients with an *HFE* mutation, neither carrying this mutation nor the *HMOX1* polymorphism was associated with radiographic joint damage, and the same was true after adjustment for well-known factors associated with arthropathy. In conclusion, this study does not support the hypothesis that *HFE* mutations or *HMOX1* polymorphisms can be used to predict the development of haemophilic arthropathy.

## 1. Introduction

The treatment landscape in haemophilia is evolving rapidly [1,2]. For years, the only treatment consisted of substituting the deficient clotting factor with standard half-life products. But, at present, extended half-life products, nonfactor products, and gene therapy are changing the therapeutic possibilities for haemophilia patients. With these new treatment modalities becoming available, treatments can be individualized. Treatment decision making is mainly guided by clinical characteristics like the bleeding frequency, presence of inhibitors, problems with venous access, etc., as well as economic considerations. Ideally, vulnerability to develop haemophilic arthropathy (HA) should also be taken into account, as joint damage is heterogenic even in patients with a similar bleeding pattern [3], and the subsequent limitations severely impact quality of life [4]. However, at present, we are unable to predict who will develop a rapidly progressive arthropathy.

HA results from repeated joint bleeds or even a single joint bleed, but haemophilia-specific joint changes are also observed in patients without overt joint bleeding [5,6,7]. Moreover, joint damage in haemophilia shows bleeding-independent progression, especially in on-demand patients [8]. HA shows degenerative features comparable to osteoarthritis, as well as inflammatory changes comparable to rheumatoid arthritis [9]. The local response to (sub)clinical bleeding and ongoing (inflammatory) responses after a bleed differ [3,10] and might be affected by a genetic predisposition [11]. 

Iron has a pivotal role in the pathogenesis of HA [12,13,14]. Upon a joint bleed, heme-derived iron accumulates as hemosiderin within the synovium, where it induces inflammation and cell proliferation, causing chronic synovitis and synovial hyperplasia [15,16,17]. Moreover, iron catalyzes the conversion of hydrogen peroxide, produced by activated chondrocytes, into hydroxyl radicals, leading to chondrocyte apoptosis [12]. Iron affects fibroblast growth factor 23 and SRY-box 9 expression, causing cartilage degeneration in HA [18]. Also, subchondral bleeding is suggested to contribute to HA development by affecting subchondral bone health and probably inducing subchondral cyst formation [19]. These processes ultimately result in the development of HA.

The body has several protective mechanisms against the damaging effects of iron and heme. Hepcidin, the expression of which is regulated by *HFE* (high iron (Fe)), inhibits oral iron absorption [20]. Genetic mutations in *HFE* are common among the Caucasian population [21] and are associated with iron overload and the development of osteoarthritis with some features resembling HA [22,23,24,25]. Cases of arthropathy in haemochromatosis and haemophilia alike are characterized by cartilage degeneration, subchondral bone changes with osteophyte and cyst formation, and osteoporosis. Also, in both disorders, synovial inflammation and proliferation occur, although this is much more explicit in haemophilia. A small-cohort study suggests that *HFE* mutations affect the severity of HA [26]. In this cohort of 34 haemophilia patients, carrying an *HFE* mutation was associated with arthropathy severity. 

Not only iron itself but also heme is tissue-destructive by inducing oxidative and inflammatory stress and providing a source of iron. Heme is broken down via a cascade of different enzymatic steps, with heme-oxygenase 1 (HO-1) being the rate-determining factor. The level of HO-1 is influenced by chemical and physical stimuli. The intensity of the HO-1 response is affected by the length of a guanine–thymidine (GT)_n_-repeat polymorphism in the promoter region of *HMOX1* (the abbreviation used for heme-oxygenase), the gene encoding HO-1 [27]. People with homozygosity for a short (GT)_n_-repeat (n-repeat < 25) have a higher response in HO-1 levels, and therefore more rapid heme degradation. Polymorphisms in *HMOX1* are associated with susceptibility to rheumatoid arthritis (RA) [28] and the progression of joint damage in RA patients [29]. 

Considering the importance of heme-derived iron in the pathogenesis of HA [22], and the suggested role of *HFE* and *HMOX1* on HA [26] and RA [28,29] development and progression, we hypothesized that a genetic predisposition for impaired iron or heme handling might impact the severity of blood-induced joint damage. To test this hypothesis, we conducted a large cross-sectional study in patients with severe and moderate haemophilia A and B.

## 2. Materials and Methods

### 2.1. Patients and Data Collection

Patients with severe or moderate haemophilia A or B (factor VIII/IX activity ≤ 5%), aged 18 years and older, and regularly visiting the outpatient clinic of the Van Creveld-kliniek, University Medical Center Utrecht (UMCU), the Netherlands, were included after providing written informed consent. The study was approved by the Institutional Review Board of the UMCU (NL42808.041.12) and performed in accordance with the Declaration of Helsinki.

Outcome was the severity of joint damage in knees, elbows, and ankles on X-rays quantified using the Pettersson score, which assesses osteochondral changes in HA in elbows, knees, and ankles (range: 0–13 points/joint and 0–78 points/patient; optimal score: 0) [30]. Blood was drawn during regular visits. From the patients’ medical files, demographic data were collected, as well as information on clotting factor activity, history of clinically relevant inhibitors (defined as a current or historic high-titer inhibitor (≥5 Bethesda Unit (BU)) or a long-term inhibitor (≥1 year and ≥1 BU)), bleeding pattern, current treatment (prophylaxis or on demand), clotting factor consumption (amount of clotting factor per kg per year), and the age at entry into the Van Creveldkliniek. Clotting factor consumption was calculated as the mean annual factor VIII/IX consumption per kilogram bodyweight over all treatment years registered from 1 January 2006 to 1 July 2016 with the exclusion of clotting factor consumption used for surgical interventions. The annualised joint bleeding rate (AJBR) was calculated as the mean number of joint bleeds per year in the wrists, elbows, shoulders, hips, knees, and ankles, using all data available from 1995 (first data in E-diary) to April 2015, independent of treatment received. 

### 2.2. Pettersson Score

All radiographs were obtained in standard care according to standard procedures and scored according to the Pettersson method [30] with the use of a reference atlas [31] by a single rater (WF) with experience using the Pettersson score. The most recent set of radiographs (elbows, knees, and ankles) taken before inclusion was used (mean interval of 5.8 years between X-rays and inclusion). The age at which the radiographs were taken was called ‘age at evaluation’. On posterior–anterior and lateral X-rays, each joint was scored for osteoporosis, epiphyseal enlargement, irregularity of subchondral surface, narrowing of joint space, subchondral cysts, erosions at joint margins, incongruence between joint surfaces, and the angulation and/or displacement of articulating bone ends. The maximum score per joint was 13, and a maximum total score per patient was 78. Joints with a prosthesis or after arthrodesis were assigned 13 points.

### 2.3. DNA Analysis

Genomic deoxyribonucleic acid (DNA) was extracted from ethylenediaminetetraacetic-acid (EDTA)-preserved blood according to a standard protocol using the Chemagic Magnetic Separation Module 1′ (MSM1) DNA extraction robot. 

The presence or absence of an *HFE* mutation was analysed at the DNA diagnostic laboratory of the UMCU. Exon 2 and 4 were amplified in a final volume of 25 μL containing 12.5 μL AmpliTaq Gold 360, 5 μL DNA at a concentration of 20 ng/μL, and a primer mixture, as outlined in Table 1. Amplification was performed on a Gene Amp 9700 thermal cycler (Applied Biosystems, Waltham, MA, USA) using an initial denaturation step at 95 °C for 10 min, followed by 33 cycles at 95 °C for 1 min, 60 °C for 1 min, 72 °C for 1 min, and a final extension step for 10 min at 72 °C. The amplified fragments were sequenced to detect the presence of c.845G>A (Cys282Tyr) or c.187C>G (His63Asp) using an ABI Prism 3700 DNA analyser (Applied Biosystems). 

As an indicator of HO-1 expression, the (GT)_n_-repeat in the promoter region of the *HMOX1* gene was established at the DNA diagnostics laboratory of the Radboud University, Nijmegen, the Netherlands. The 5′-flanking region containing (GT)_n_-repeats of the HO-1 gene was amplified via polymerase chain reaction (PCR) using an FAM-labeled sense primer, 5′-TCACAGGTCAGTTGTAGGGATG-3′, and an unlabeled antisense primer, 5′-ACAGCTGATGCCCATTTCT-3′. PCR products were generated in a final volume of 10 μL, containing one unit AmpliTaq Gold DNA polymerase (Applied Biosystems, Nieuwekerk a/d IJssel, The Netherlands), AmpliTaq PCR buffer II, 2.5 mM MgCl_2_, 0.25 mM of each dNTP, 5 pmoles of each primer, and 50 ng genomic DNA. After a 10 min denaturation period at 94 °C, PCR products were amplified in 35 cycles of 94 °C for 1 min, 62.4 °C for 1 min, and 72 °C for 1 min. A final extension step of 10 min at 72 °C completed the reaction. One μL of each amplified product (10x diluted) was combined with size standard (LIZ-600; Applied Biosystems) and formamide, and analysed using an ABI3730 Genetic Analyser (Life Technologies, Carlsbad, CA, USA).

### 2.4. Iron Status

Serum was obtained from blood collected into Vacutainer tubes (10 mL, Becton Dickinson, Oxford, UK). Samples were centrifuged at 1300× *g* for 10 min. Serum aliquots were obtained and stored at −80 °C until use. Serum ferritin was determined using an automated chemiluminescence immunoassay on a Unicel Dxl 800 (Beckman Coulter, Brea, CA, USA). Serum iron was estimated using a colorimetric assay on a DxAU 5811 (Beckman Coulter). Serum transferrin values were obtained via immunochemical turbidimetry on a DxAU 5811. Total iron binding capacity (TIBC) (μmol/L) was calculated as serum transferrin (g/L) × 25.14, and transferrin saturation as the ratio of serum iron to TIBC. As a marker for inflammation, C-reactive protein (CRP) was measured via immunochemical turbidimetry on a DxAU 5811.

### 2.5. Statistical Analysis

Statistical analyses were performed using IBM SPSS Statistics 26.0.0 for Windows (IBM, Chicago, IL, USA). Comparison between two groups was performed via independent t-test, after ln-transformation if necessary, to achieve normal distribution. Comparisons between three groups were performed via one-way ANOVA with post hoc Bonferroni to correct for multiple testing. Nominal data were analysed via the Pearson chi-squared test. *p*-values <0.05 were considered statistically significant.

According to the *HFE* mutation, patients were allocated into two groups:

- “Wild type”(WT), including patients without a Cys282Tyr or His63Asp mutation;
- “Mutated”, including patients heterozygous for Cys282Tyr and/or His63Asp, or homozygous for His63Asp or Cys282Tyr.
- For the *HMOX1* polymorphism, patients were allocated into three groups:
- Patients homozygous for a (GT)_n_-repeat < 25, referred to as SS (S for short);
- Patients homozygous for a (GT)_n_-repeat ≥ 25, referred to as LL (L for long);
- Patients heterozygous for a (GT)_n_-repeat ≥ 25, referred to as SL.

Linear regression analysis was performed to assess the association of the *HFE* mutation and *HMOX1* polymorphism with the Pettersson score. We performed a stepwise linear regression to include all potential confounders of the relationship between the genetic mutations and the Pettersson score. Covariates investigated were haemophilia type and severity, AJBR, current treatment (prophylaxis versus on demand), clotting factor consumption, presence of clinically relevant inhibitors (for definition, see ‘patients and data collection’), age at evaluation (age at moment of X-rays), age at entry into the Van Creveld-kliniek, and year of birth. Patients were grouped in three cohorts according to their year of birth (≤1965; 1966–1980; ≥1981), as access to treatment significantly improved over the years (in the early 1960s, replacement therapy with plasma-derived clotting factors became available; in the early 1980s, prophylactic treatment became possible [32]). For the age at entry into the Van Creveldkliniek, patients were allocated to two groups with a cut off at 4 years of age, as this was also assumed to impact access to treatment.

Factors with a *p*-value < 0.10 were considered significant confounders and included in the model. Model assumptions were checked using a Q-Q plot and normality of the residuals was checked with a normal P-P plot. 

## 3. Results

### 3.1. Patient Characteristics

In our cross-sectional study, 252 patients with severe and moderate haemophilia regularly visiting the Van Creveldkliniek, University Medical Center Utrecht, the Netherlands, were included. The mean age was 44 years (range: 18–79), and the majority had severe haemophilia A (185 of 252; 73%); see Table 2. The median annualized joint bleeding rate (AJBR) was 2.3 (IQR 1.0–4.6) for patients with severe haemophilia versus 0.5 (IQR 0.1–2.2) for moderate haemophilia (*p* = 0.00), and the median clotting factor consumption was 1851 IU/kg/y (IQR 1143–2341) versus 236 IU/kg/y (IQR 69–1793) (*p* = 0.00), respectively. 

Outcome was the severity of joint damage in knees, elbows, and ankles on X-rays, quantified using the Pettersson score with a median of 22 (IQR 6–44) for severe haemophilia versus 4 (IQR 1–13) for moderate haemophilia (*p* = 0.00, unadjusted for age at evaluation). 

### 3.2. HFE Mutation

The presence of an *HFE* mutation was detected in 38% of the patients (n = 79 patients with severe haemophilia; n = 16 patients with moderate haemophilia); one patient was found to be homozygous for the Cys282Tyr mutation, and six patients were compound heterozygous for Cys282Tyr and His63Asp (frequency similar to the frequency observed in the Northern European population [21]). Wild-type (WT) patients were comparable to patients carrying a mutation in terms of age at time of evaluation, haemophilia type or treatment, AJBR, or Pettersson score. The clotting factor consumption was slightly higher in patients carrying a mutation (1.5 (0.6–2.2) vs. 1.8 (1.0–2.2) 1000 IU/kg/y; *p* = 0.03), but this difference was not considered clinically important. In patients with an *HFE* mutation, plasma transferrin saturation and iron levels were increased (transferrin saturation 27% vs. 32%; *p* = 0.01, iron 17 vs. 19 μmol/L; *p* = 0.04). A transferrin saturation exceeding 45% (the cut-off used for suspicion of haemochromatosis) was detected in six (4%) WT patients compared to 13 (14%) patients carrying a mutation (*p* = 0.01). In addition, ferritin showed a trend towards increased levels in the mutated group (median 98 (IQR 58–157) vs. 86 (IQR 54–133) μg/L; *p* = 0.06), whereas CRP levels were similar (median 2.1 (IQR 0.6–4.9) vs. 1.5 (IQR 0.5–3.3) mg/L; *p* = 0.16). Ferritin levels of >250μg/L were observed in five (3%) WT patients versus 10 (11%) in the mutated group (*p* = 0.02). 

### 3.3. HMOX1 Polymorphism

Homozygosity for the short (GT)_n_-repeat (SS) was found in 22 patients (n = 16 with severe haemophilia), 116 patients were homozygous for the long (GT)_n_-repeat (LL) (n = 96 with severe haemophilia), and 113 patients were heterozygous (SL) (n = 99 with severe haemophilia). The *HMOX1* polymorphism could not be determined in one patient due to technical issues. Patients in the SL cohort more frequently had haemophilia A compared to SS and LL patients (95% vs. 77% and 84%, respectively; *p* = 0.012). SS patients had a lower AJBR compared to SL patients (1.3 (0–3.4) vs. 2.3 (1.0–4.2); *p* = 0.02), whereas there was no difference between SS and LL patients (1.3 (0–3.4) vs. 2.0 (0.5–4.6); *p* = 0.08). Other baseline characteristics were similar between the three cohorts.

### 3.4. Association of HFE Mutation or HMOX1 Polymorphism with Haemophilic Arthropathy

The association between the genetic polymorphisms and radiographic joint damage was investigated via linear regression analysis. In this analysis, 248 patients were included; three patients were excluded because of the usage of a bypassing agent; in one patient, the *HMOX1* polymorphism was unknown. Factors independently associated with the Pettersson score were the severity of haemophilia, age at time of evaluation, access to clotting factor replacement (defined as birth cohort and age of entry into the Van Creveldkliniek), clotting factor consumption, and the AJBR (all *p* < 0.05; Table 3). There was no association between *HFE* and *HMOX1* and the Pettersson score without adjustment for other factors (unstandardized beta for *HFE* 3.5; *p* = 0.24, for *HMOX1* SS vs. SL and LL −2.1; *p* = 0.27). Data were similar according to haemophilia type (A versus B, unstandardized beta −0.60; *p* = 0.89), current prophylactic treatment (yes or no, unstandardized beta −5.12; *p* = 0.10), and inhibitor status (unstandardized beta 4.50; *p* = 0.23). The r-square of the linear regression model including all significantly associated factors was 0.63; the addition of the *HFE* and *HMOX1* data to this model did not improve this (Table 3). 

## 4. Discussion

The treatment of haemophilia aims to prevent joint bleeding and subsequent joint damage. With new treatment modalities becoming available, personalized medicine is becoming more and more possible. Being able to predict an individual’s vulnerability to developing HA could guide treatment decisions. This is the first study specifically designed to investigate the association between the severity of HA and the genetic polymorphisms influencing iron and heme homeostasis. In this cohort, the severity of HA represented by the Pettersson score was not associated with *HFE* mutations or the (GT)_n_-repeat in the promotor region of the *HMOX1* gene, even after adjustment for well-known factors impacting the development of HA. Although carrying an *HFE* mutation significantly increased serum iron levels and transferrin saturation, this did not result in increased joint damage reflected on X-rays.

Our study contradicts the role of *HFE* in the severity of HA that was suggested by Cruz et al. [26]. Their study had several limitations; the population size was small, the AJBR in patients with severe haemophilia was high, joint damage was subjectively defined as the number of affected joints and number of haemarthroses per year, and treatment data were not included. In our study, carrying an *HFE* mutation led to increased serum levels of iron and transferrin saturation, but this did not result in a difference in Pettersson score, AJBR, or number of affected joints. 

In contrast to the role of *HMOX1* polymorphisms in the susceptibility to RA and subsequent inflammation [28,29], our study showed no relation with HA severity. To objectively assess arthropathy, we used plain radiographs of the ankles, knees, and elbows, and scored them according to the Pettersson scoring method, as this is a widely used method to quantify osteochondral changes with a good reliability and agreement. The limitations are its insensitivity for early small changes and low sensitivity for soft-tissue changes. As such, a possible association with soft-tissue changes including inflammation might be missed. However, as soft-tissue changes will ultimately result in osteochondral changes and the median Pettersson score was 17 (IQR 4–39) in our cohort, this was an adequate tool to assess HA severity in this study. Future studies using ultrasound to investigate soft-tissue changes in young patients with early prophylaxis might be interesting to further elucidate potential factors affecting response to bleeding and vulnerability to developing joint damage.

Another potential limitation is the age range in our cohort including differences in treatment over time. To address this, we grouped patients according to their date of birth with cut-offs at landmarks in the history of haemophilia treatment (1965 and 1980). Subdividing patients born between 1965–1980 and ≥1980 did not affect the regression model. Ideally, studying the impact of genetic variations is performed prospectively in a homogenous population with similar treatment, bleeding frequency, and assessment of joint damage at specific time points. However, it will still remain difficult to investigate the impact of a single factor on joint damage progression, as HA is a cumulative result of bleeds, treatment, joint loading, exercise, etc., developing over years, with many of these factors being difficult to measure. Our study was performed in a unique large single-centre cohort with a complete set of detailed data on treatment and bleeding without interference from centre-specific treatment guidelines.

In conclusion, this cross-sectional cohort study does not support the hypothesis that *HFE* mutations or *HMOX1* polymorphisms are associated with the severity of HA. 

## Figures and Tables

**Table 1:** Primers used for polymerase chain reaction to detect *HFE* mutations.

Forward	5′-*GTTTTCCCAGTCACGAC*CTGCAACTCACCCTTCACAA-3′
Reverse	5′-CAACAGTGAACATGTGATCCC-3′
Forward	5′-GGTCTTTCCTTGTTTGAAGCTT-3′
Reverse	5′-*GTTTTCCCAGTCACGAC*AAATTCCTTCCCTCTTCCCTG-3′
Forward	5′-ATAGAAGGAAGTGAAAGTTCCAGTC-3′
Reverse	5′-*GTTTTCCCAGTCACGAC*CAAGGTTATCCAGCCCTGGTA-3′
Forward	5′-*GTTTTCCCAGTCACGAC*GTGTCGGGCCTTGAACTAC-3′
Reverse	5′-CATAATTACCTCCTCAGGCACTC-3′

In *italic* is the M13 sequence used as the universal tail for sequencing.

**Table 2:** Baseline characteristics according to haemophilia severity.

	Severe Haemophilia n = 211	Moderate Haemophilia n = 41	*p*
Age at inclusion, mean (range)	44 (18–79)	44 (18–75)	0.95
Age at evaluation, mean (range)	37 (11–79)	42 (15–75)	0.05
Haemophilia A, n (%)	185 (88%)	38 (93%)	0.44
Inhibitor history, n (%)	26 (12%)	1 (2%)	0.09
Currently on prophylaxis, n (%)	165 (78%)	3 (7%)	0.00
Clotting factor consumption (1000 IU/kg/y), median (IQR)	1.9 * (1.1–2.3)	0.2 (0.07–1.8)	0.00
AJBR, median (IQR)	2.3 (1.0–4.6)	0.5 (0.1–2.2)	0.00
Pettersson score, median (IQR)	22 (5–44)	4 (1–13)	0.00

AJBR, annualised joint bleeding rate; IQR, interquartile range; SD, standard deviation. * n = 208; three patients excluded because of using bypassing agents.

**Table 3:** Multivariate linear regression analysis for determinants of Pettersson score.

Predictor	Regression Coefficient	95% CI	*p*
Age at evaluation (per y)	0.48	0.29–0.67	0.00
Clotting factor activity (severe vs. non-severe)	−16.2	−21.4–−10.8	0.00
Clotting factor consumption (per 1000/IU/kg/y)	3.4	1.5–5.3	0.00
AJBR (per bleed)	1.0	0.5–1.6	0.00
Year of birth (≤1965 vs. later)	−15.0	−20.2–−9.8	0.00
Entry into the clinic (≤age 4 y or later)	6.6	2.4–10.8	0.00
*HFE* mutation	1.0	−2.7–4.6	0.60
HMOX1 polymorphism SL	1.8	−4.7–8.3	0.59
LL	0.3	−6.1–6.8	0.92

AJBR, annualised joint bleeding rate.

## Data Availability

Data are available from the first author (L.F.D. van Vulpen, l.f.d.vanvulpen-2@umcutrecht.nl) upon request.
